# Ultrasound-Guided Obturator Nerve-Targeted Physical Therapy for Persistent Buttock Pain After Hip Arthroscopy for Femoroacetabular Impingement: A Report of Two Cases

**DOI:** 10.7759/cureus.95021

**Published:** 2025-10-21

**Authors:** Masayoshi Saito, Masashi Kawabata, Shunta Shimizu, Toru Omodani

**Affiliations:** 1 Department of Rehabilitation, Tokyo Advanced Orthopaedics, Tokyo, JPN; 2 Department of Rehabilitation, Kitasato University School of Allied Health Sciences, Sagamihara, JPN; 3 Department of Orthopaedics, Tokyo Advanced Orthopaedics, Tokyo, JPN

**Keywords:** external obturator muscle, faber test, femoroacetabular impingement, hip arthroscopy, obturator nerve, ultrasound-guided hydrodissection

## Abstract

Femoroacetabular impingement (FAI) can present with buttock pain even after hip arthroscopy. While arthroscopy often improves pain and function, some patients continue to experience persistent postoperative symptoms.

Case 1: A woman in her 40s presented with buttock pain specifically induced during walking, eight months after arthroscopy for a labral tear. The FABER (Flexion, Abduction and External Rotation) test reproduced buttock pain (numerical rating scale (NRS) 5), with increased knee-to-floor distance (KFD), poor external obturator (EO) contraction, and localized tenderness elicited under ultrasound-guided sonopalpation of the obturator nerve (ON), EO, and internal obturator.

Case 2: A man in his 30s reported severe buttock pain during hip abduction-external rotation 11 months after arthroscopy. The FABER test reproduced pain (NRS 7), with increased KFD, diminished EO contraction, and tenderness around the ON and EO on ultrasound-guided sonopalpation.

Both patients underwent weekly ultrasound-guided manual therapy with EO activation and biweekly ON hydrodissection using 10 mL of 0.09% lidocaine solution, followed by dynamic stretch-slack maneuvers.

Both demonstrated immediate improvement in FABER-induced buttock pain, shortened KFD, and enhanced EO contraction after each session. In Case 1, buttock pain during walking resolved after the fourth session without recurrence. In Case 2, progressive improvement began at the second session, with complete recovery of daily activities.

These cases suggest that impaired gliding of ON articular branches may contribute to postoperative buttock pain after hip arthroscopy. Ultrasound-guided ON hydrodissection with EO activation and targeted nerve-gliding maneuvers may offer an effective therapeutic approach. Limitations include the small number of cases, qualitative evaluation without validated outcome scores, and short-term follow-up.

## Introduction

Femoroacetabular impingement (FAI) has been increasingly recognized as a cause of hip and groin pain [1], with arthroscopic surgery widely performed to restore function and alleviate symptoms [2]. Although many patients experience improvement, persistent postoperative pain remains a global concern, with systematic reviews reporting suboptimal patient satisfaction and delayed return to sport in a substantial proportion of cases [3].

Recent evidence indicates that return-to-sport rates after hip arthroscopy, while generally high, are not universally complete. For example, a systematic review reported a pooled return-to-sport rate of 93% at any level and 82% at the preoperative level [4]. Another recent review found return rates ranging from 75.6% to 98%, depending on the population studied [5], suggesting that incomplete recovery and residual functional limitations remain a challenge. These findings underscore that, despite the relative success of hip arthroscopy, outcomes are not uniformly favorable, and a subset of patients continue to experience pain and reduced quality of life for years after surgery [6].

While persistent groin pain after hip arthroscopy has been investigated, buttock pain and its potential relation to obturator nerve (ON) dysfunction remain underrecognized. The ON provides articular branches to the hip joint and runs between the external and internal obturator muscles, placing it at risk of entrapment or impaired gliding. Irritation of these branches may produce buttock or medial thigh pain, yet ON dysfunction has been largely underexplored in the literature.

To address this gap, we report two cases of patients with refractory buttock pain after hip arthroscopy, in whom ultrasound-guided assessment identified ON- and external obturator (EO)-related tenderness, and targeted interventions including ON hydrodissection and EO activation provided symptom relief. Although we cannot exclude the possibility of prior similar reports, to the best of our knowledge, descriptions of ON dysfunction after hip arthroscopy are scarce, and no previous reports have specifically described the combined use of ON hydrodissection and EO activation for refractory postoperative buttock pain. This report highlights the potential clinical relevance of ON dysfunction in post-arthroscopy pain and underscores the need for further research in this area.

## Case presentation

Case 1

**Figure 1:**
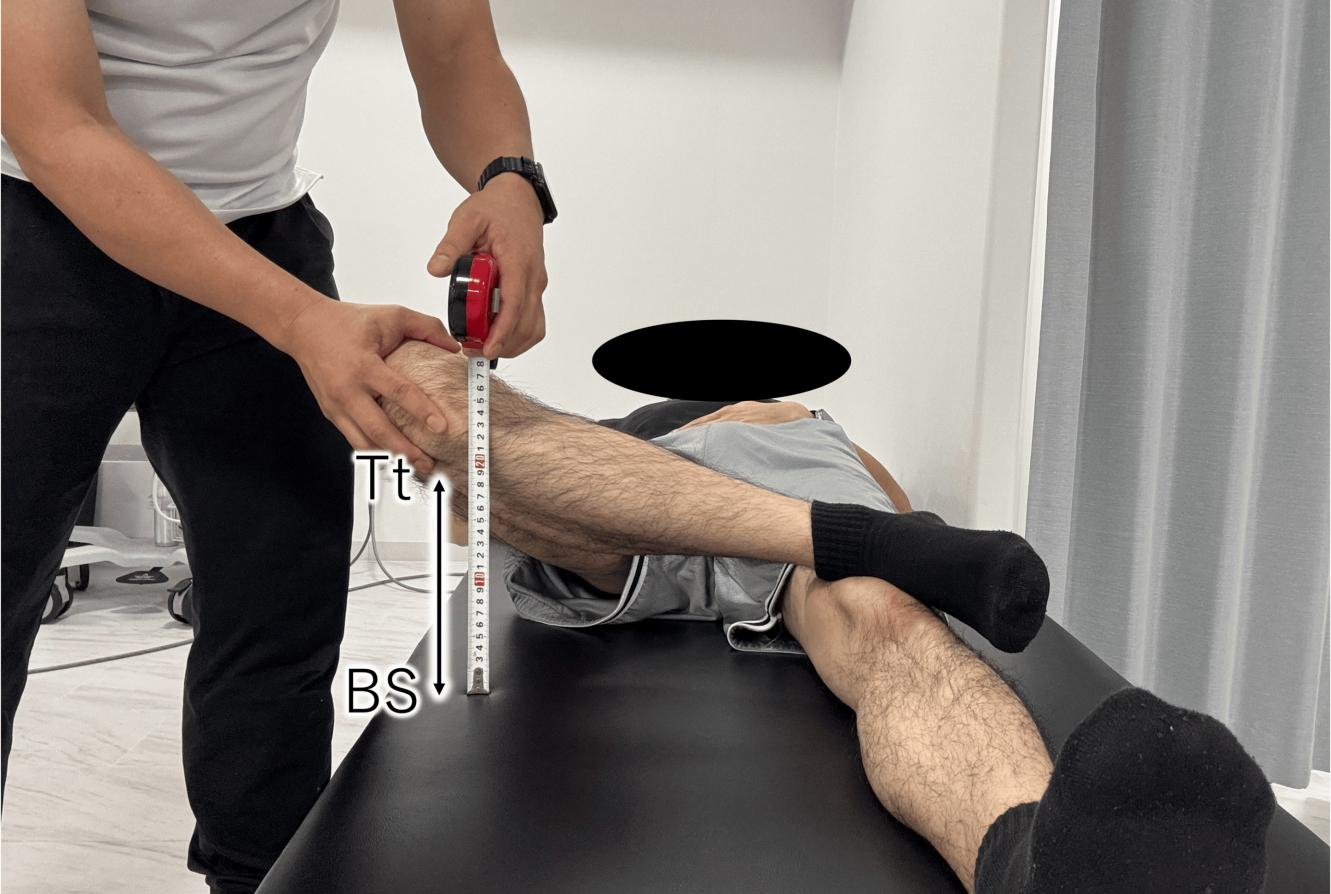
Measurement of knee-to-floor distance (KFD) in the FABER position In an illustrative model, the subject is positioned supine with the tested hip in flexion, abduction, and external rotation (FABER) position. The vertical distance between the lateral aspect of the knee and the floor is measured using a ruler. Tt, Tibial tuberosity; BS, Bed surface

A woman in her 40s presented with buttock pain specifically induced during walking, eight months after undergoing right hip arthroscopy for a labral tear. Prior intra-articular injections, platelet-rich plasma therapy, and sciatic nerve hydrodissection had failed to relieve symptoms. At the initial evaluation, the alexion, abduction, and external rotation (FABER) test reproduced buttock pain (numerical rating scale (NRS) 5). The knee-to-floor distance (KFD) (illustrative model: Figure 1) [7] was 35.5 cm on the symptomatic side compared with 26.5 cm contralateral. Manual muscle testing of hip external rotation (side-lying, hip flexed to 90°, with resisted internal rotation) was graded as two, as the patient was unable to maintain the test position against gravity. Ultrasound imaging revealed poor EO contraction, and ultrasound-guided sonopalpation elicited marked tenderness around the ON, EO, and internal obturator muscle. No relevant comorbidities or medications were reported. Based on these findings, ON entrapment and EO dysfunction were suspected.

Case 2

A man in his 30s presented with buttock pain during hip abduction-external rotation and discomfort posterior to the greater trochanter, 11 months after left hip arthroscopy for a labral tear. Intra-articular injections proved ineffective. The FABER test elicited severe pain (NRS 7) with increased KFD [7] on the symptomatic side (31.0 cm vs. 24.5 cm contralateral). Hip external rotation strength was likewise graded as 2 on manual testing, consistent with the findings in Case 1. Ultrasound imaging revealed poor EO contraction, and ultrasound-guided sonopalpation revealed localized tenderness around the ON and EO. He had no significant medical history, was not taking regular medication, and had not undergone prior structured physical therapy. These findings suggested possible ON involvement and impaired EO function.

Intervention

**Figure 2:**
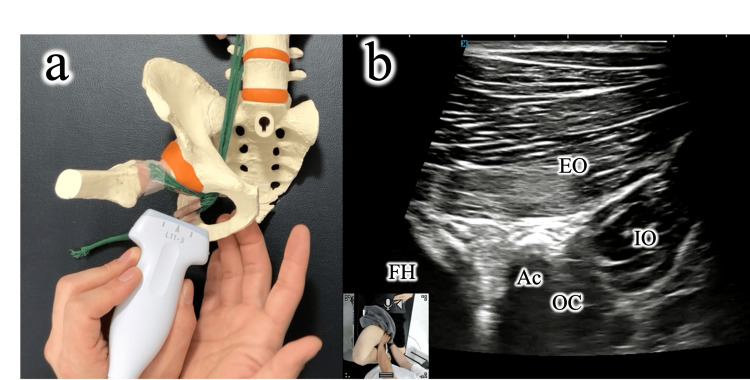
Short-axis gliding manipulation of the obturator nerve Under ultrasound guidance, long-axis views of the external obturator muscle and obturator canal are obtained. A gliding maneuver is applied to the obturator nerve between the external and internal obturator muscles. (a) Schematic illustration created by the author. (b) Ultrasound-guided manual technique. EO, External obturator muscle; IO, Internal obturator muscle; OC, Obturator canal; Ac, Acetabular; FH, Femoral head.

Both patients underwent weekly manual therapy and exercises, combined with biweekly ultrasound-guided ON hydrodissection. During manual therapy, the long-axis view of the EO muscle and the obturator canal was visualized under ultrasonographic guidance. Ultrasound guidance was essential to ensure accurate identification of the obturator nerve and its surrounding structures, which cannot be reliably localized by palpation alone. This allowed precise application of manipulation as the nerve passed between the external and internal obturator muscles. With the examining fingers advanced from the inferior pubic and ischial rami, gliding manipulation was applied to the ON as it passed between the external and internal obturator muscles. This procedure is illustrated in an anatomical model (Figure 2) and demonstrated in Videos 1, 2.

**Video 1:** Short-axis gliding manipulation of the obturator nerve (schematic illustration) A gliding maneuver is applied to the obturator nerve between the external and internal obturator muscles.

**Video 2:** Short-axis gliding manipulation of the obturator nerve (ultrasound-guided manual technique) In an illustrative model, a long-axis view of the EO muscle and the obturator canal (OC) is obtained under ultrasound guidance. A gliding maneuver is then applied to the ON between the EO and IO muscles. EO, External obturator muscle; IO, Internal obturator muscle; OC, Obturator canal; Ac, Acetabular; FH, Femoral head

**Figure 3:**
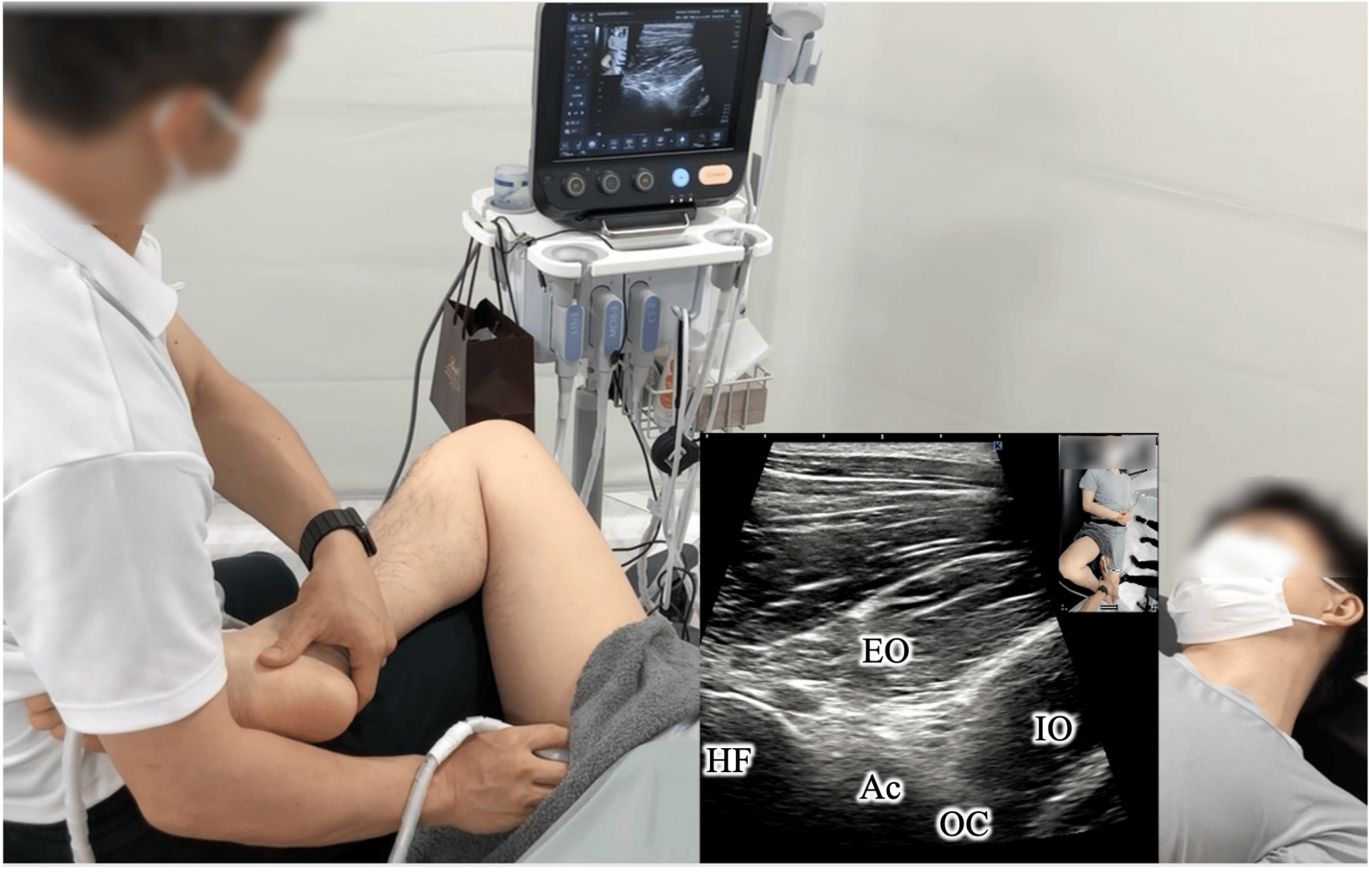
Activation of the external obturator (EO) muscle In an illustrative model, real-time ultrasound visual feedback is employed to repeatedly facilitate EO contraction until tenderness diminishes. EO, External obturator muscle; IO, Internal obturator muscle; OC, Obturator canal; Ac, Acetabular; FH, Femoral head

Therapeutic exercises targeted EO contraction using real-time ultrasound visual feedback, repeated until the muscle response improved and local tenderness decreased (illustrative model: Figure 3; Video 3).

**Video 3:** Activation of the external obturator muscle In an illustrative model, real-time ultrasound visual feedback is employed to repeatedly facilitate external obturator muscle contraction until tenderness diminishes. EO: External obturator muscle, IO, Internal obturator muscle; Ac, Acetabular; FH, Femoral head

**Figure 4:**
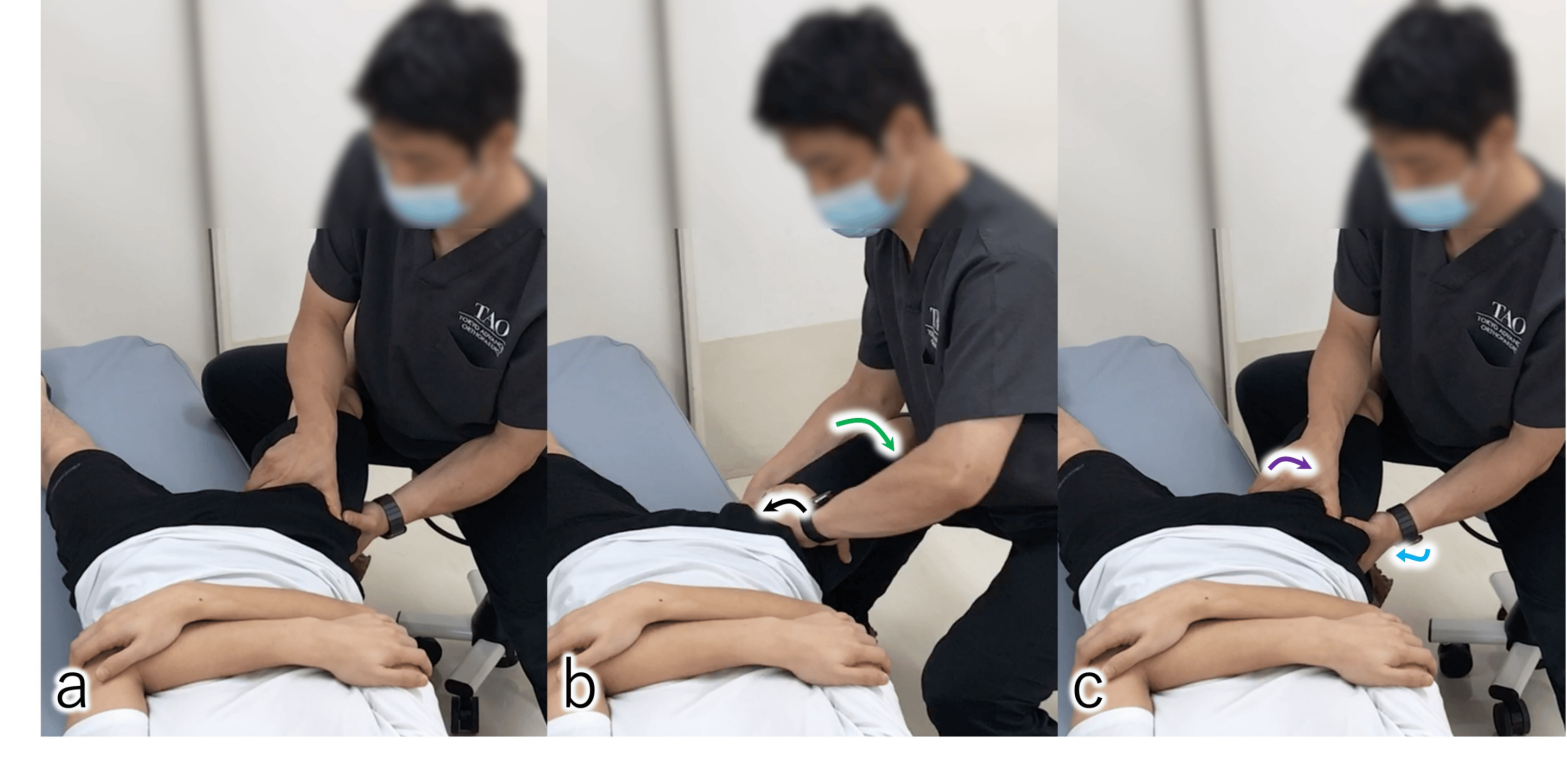
Long-axis gliding and stretching of the obturator nerve (ON) In the supine position, internal hip rotation (black arrow) with abduction (green arrow) stretches the ON, followed by external rotation (purple arrow) with adduction (blue arrow) to slacken the nerve. This stretch–slack sequence is repeated until nerve mobility improves and resistance diminishes. (a) Starting position; (b) stretch position, (c) slack position.

Hydrodissection of the ON was performed within the deep layer of the EO muscle using 10 mL of 0.09% lidocaine diluted in saline. Immediately after each injection, longitudinal nerve gliding was promoted using dynamic stretch-slack maneuvers [8]: supine hip internal rotation with abduction (stretch), followed by external rotation with adduction (slack). This stretch-slack sequence was repeated until the resistance during joint motion was alleviated, demonstrated in an illustrative model (Figure 4; Video 4).

**Video 4:** Long-axis gliding and stretching of the obturator nerve In the supine position, an illustrative model demonstrates internal hip rotation with abduction stretches the obturator nerve, followed by external rotation with adduction to slacken the nerve. This stretch-slack sequence is repeated until nerve mobility improves and resistance diminishes.

Outcome

In both patients, immediate improvements were observed after each session of ultrasound-guided hydrodissection and exercise therapy (Case 1: Video 5, Pre-treatment; Video 6, Post-treatment; Case 2: Video 7, Pre-treatment; Video 8, Post-treatment).

**Video 5:** External obturator muscle contraction before manual and exercise therapy in Case 1 EO: External obturator muscle, IO, Internal obturator muscle; Ac, Acetabular; FH, Femoral head

**Video 6:** External obturator muscle contraction after manual and exercise therapy in Case 1 EO: External obturator muscle, IO, Internal obturator muscle; Ac, Acetabular; FH, Femoral head

**Video 7:** External obturator muscle contraction before manual and exercise therapy in Case 2 EO: External obturator muscle, IO, Internal obturator muscle; Ac, Acetabular; FH, Femoral head

**Video 8:** External obturator muscle contraction after manual and exercise therapy in Case 2 EO: External obturator muscle, IO, Internal obturator muscle; Ac, Acetabular; FH, Femoral head

The buttock pain induced by the FABER test was markedly reduced, the KFD was shortened, and external rotation strength improved (Table 1).

**Table 1:** Overview of clinical findings and therapeutic course FABER, Flexion, Abduction, and External Rotation; NRS, Numerical Rating Scale

	Parameter	1stsession	2ndsession	3rdsession	4thsession
	Time point	(Week 0)	(Week 1)	(Week 2)	(Week 4)
Patient 1	Pain during FABER test (NRS: 0-10)	5	3		0
	Knee-to-floor distance (cm)	26.8	23.5	22	22
	External obturator muscle (grade: 0-5)	2	3	4	4
	Pain during walking	+	+	+	―
Patient 2	Pain during FABER test (NRS: 0-10)	7	2	2	
	Knee-to-floor distance (cm)	21	20	20	
	External obturator muscle (grade: 0-5)	2	3	4	
	Pain during walking	+	+	―	

In Case 1, the walking-related buttock pain was resolved completely by the fourth treatment session, with no recurrence observed thereafter. In Case 2, gradual improvement was noted from the second session onward, with full resolution of daily activity limitations.

At the latest follow-up, more than six months after the final intervention, no recurrence of buttock pain was observed in either case.

## Discussion

Hip arthroscopy is widely performed for FAI and generally provides good pain relief and functional improvement. Nevertheless, a subset of patients experience persistent postoperative pain and delayed recovery, suggesting that factors beyond intra-articular pathology - such as extra-articular or neuromuscular dysfunction - may contribute to ongoing symptoms.

In the present cases, both patients developed buttock pain after hip arthroscopy, which was provoked during the FABER test and walking, suggesting a component of extra-articular dysfunction. Ultrasound imaging revealed poor contraction of the EO, and ultrasound-guided sonopalpation elicited tenderness along the ON. Based on the clinical findings and pain distribution, ON involvement was suspected as a potential pain generator.

**Figure 5:**
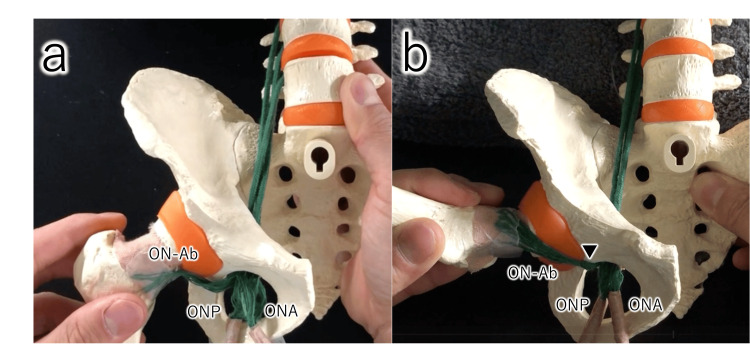
Course of the obturator nerve (ON) articular branches (illustrated model created by the author) After passing through the obturator canal, the articular branches of the ON abruptly change direction before terminating in the hip capsule. (a) Neutral position; (b) Flexion, ABduction, and External Rotation (FABER) position ON-Ab, Articular branch of the obturator nerve; ONA, Anterior branch of the obturator nerve; ONP, Posterior branch of the obturator nerve. ▼: Point where the articular branch of the obturator nerve abruptly changes course.

Biomechanically, the FABER position tensions the anteroinferior hip capsule innervated by the ON articular branches [9]. Kampa et al. reported that ON branches innervate the 3 o’clock and 6:30 clock position in the region of the hip capsule [10]. Therefore, the ON innervation extends not only to the anterior hip but also to the buttock and posterior thigh. After traversing the obturator canal, the ON articular branches abruptly change direction before reaching the capsule (Figure 5), which may predispose them to entrapment or impaired gliding.

Bassett et al. demonstrated that hip extension combined with abduction produces distal displacement of the ON [11], suggesting similar mechanics may underlie FABER-related pain. These anatomical and biomechanical relationships may explain why buttock pain was elicited during FABER testing in both cases.

Peripheral nerve-targeted interventions have been reported for peripheral nerve-related pain around the hip region, including those involving the sciatic, femoral, and pudendal nerves. In our cases, the pain was localized to the buttock rather than the anterior or perineal regions, making femoral or pudendal nerve involvement less likely. Moreover, sciatic nerve hydrodissection performed previously in Case 1 did not relieve symptoms, supporting that the ON was the more plausible source. The immediate reduction in FABER-evoked pain and shortening of knee-to-floor distance (KFD) after each session of ON hydrodissection, combined with EO activation exercises, further supports a neural-mechanical mechanism involving improved perineural mobility and muscle function.

Various techniques for ON blockade have been described, targeting sites proximal or distal to the obturator canal [12,13]. In cadaveric studies, Yoshida et al. demonstrated that an injectate administered just distal to the canal can spread to the articular branches [13]. These findings support the rationale for distal ON hydrodissection. Combining hydrodissection with gliding maneuvers and EO activation may not only enhance the pharmacological effect of the injectate but also promote nerve mobility.

The mechanism underlying symptom improvement may involve both mechanical and neuromuscular effects. Hydrodissection likely reduced entrapment and improved ON gliding, while EO reactivation may have enhanced dynamic hip stability. The observed immediate changes after injection, however, suggest that neural decompression played a principal role in pain reduction, which was subsequently maintained through targeted rehabilitation. At follow-up beyond six months after the final intervention, neither patient experienced recurrence of buttock pain, suggesting the durability of the treatment effect.

This report has several limitations. First, it includes only two cases without a control group, which limits generalizability. Second, the improvements observed could be influenced by other factors such as placebo or Hawthorne effects, natural recovery, or the effect of rehabilitation alone. Third, the multimodal nature of our approach - combining ON hydrodissection, EO activation, and exercise therapy - makes it difficult to determine the relative contribution of each component. Fourth, outcomes were assessed qualitatively without validated scales such as the Hip Outcome Score - Activities of Daily Living (HOS-ADL) or International Hip Outcome Tool-33 (iHOT-33). Fifth, the pressure applied during ultrasound-guided sonopalpation was not quantified. Finally, although pudendal nerve entrapment has been described as a rare postoperative complication, this nerve was not evaluated in the present cases. These limitations highlight the exploratory nature of the study and emphasize the need for future controlled research using standardized assessment tools and longer follow-up.

## Conclusions

Persistent buttock pain post-hip arthroscopy may involve ON. Ultrasound-guided interventions combining ON hydrodissection, manual gliding techniques, and EO muscle activation may serve as effective strategies for managing FABER-induced buttock pain in postoperative patients. To translate these findings into broader clinical practice, further research is warranted to establish clear indications.
